# Combined Cornus Officinalis and Paeonia Lactiflora Pall Therapy Alleviates Rheumatoid Arthritis by Regulating Synovial Apoptosis *via* AMPK-Mediated Mitochondrial Fission

**DOI:** 10.3389/fphar.2021.639009

**Published:** 2021-04-09

**Authors:** Lichuang Huang, Shaoqi Hu, Meiyu Shao, Xin Wu, Jida Zhang, Gang Cao

**Affiliations:** ^1^ School of Pharmacy, Zhejiang Chinese Medical University, Hangzhou, China; ^2^ College of Basic Medical Science, Institute of Basic Research in Clinical Medicine, Zhejiang Chinese Medical University, Hangzhou, China

**Keywords:** ursolic acid, paeoniflorin, inflammation, oxidative stress, synovial tissue

## Abstract

Rheumatoid arthritis (RA) is a chronic autoimmune disease that leads to cartilage destruction and bone erosion. In-depth exploration of the pathogenesis of RA and the development of effective therapeutic drugs are of important clinical and social value. Herein, we explored the medicinal value of Cornus officinalis Sieb. and Paeonia lactiflora Pall. in RA treatment using a rat model of collagen-induced arthritis (CIA). We compared the therapeutic effect of Cornus officinalis and Paeonia lactiflora with that of their main active compounds, ursolic acid and paeoniflorin, respectively. We demonstrated that the combination of Cornus officinalis and Paeonia lactiflora effectively inhibited the release of factors associated with oxidative stress and inflammation during RA, therein ameliorating the symptoms and suppressing the progression of RA. We further showed that the underlying mechanisms may be related to the regulation of apoptosis in synovial tissues, and we investigated the potential involvement of AMPK-mediated mitochondrial dynamics in the therapeutic action of the two drugs and their active components.

## Introduction

Rheumatoid arthritis (RA) is a chronic autoimmune disease that leads to cartilage destruction and bone erosion (Gong et al., 2019; Tseng et al., 2020). It usually occurs in individuals aged 30–50, and its incidence rate in women is approximately three times that in men (Intriago et al., 2019; Kohler et al., 2019). RA exhibits an intricate pathological course and is difficult to manage, and the high disability rate severely lowers the quality of life, bringing about heavy socioeconomic burdens. The pathogenesis of RA is not completely clear and may be related to a variety of factors such as genetics, environment, and immunity (Klareskog et al., 2006; Deane et al., 2017). Pathological features of RA include joint inflammation, synovial hyperproliferation, and articular swelling (Guo et al., 2018). RA progression may lead to a number of comorbid conditions such as coronary heart disease, atherosclerosis, and interstitial pneumonia (Kaplan, 2010; Kakuwa et al., 2019), resulting in significant life span shortening in some RA patients. Therefore, in-depth exploration of the pathogenesis of RA and the development of effective therapeutic drugs are of important clinical and social value.

Oxidative stress has been heavily implicated in the pathophysiology of inflammatory diseases such as RA (da Fonseca et al., 2019). As the main organelle involved in energy metabolism and a main source of intracellular reactive oxygen species (ROS), mitochondria play an indispensable role in homeostatic maintenance. Normal mitochondrial function is critical in upholding redox balance (Ippolito et al., 2020), and overproduction of ROS leads to a disruption in redox equilibrium and mitochondrial dysfunction, which may aggravate arthritic development (Valcarcel-Ares et al., 2014). In addition, excessive ROS is associated with immunopathogenesis and inflammation. For example, macrophages are activated by pro-inflammatory factors such as interleukin (IL)-1β and tumor necrosis factor (TNF)-α under oxidative stress to produce superoxides (Filippin et al., 2008), in turn accelerating the development of arthritis. The maintenance of proper mitochondrial function is important in the regulation of cell fate. In particular, mitochondrial fission has been shown to be closely linked to cell apoptosis, and the balance between mitochondrial fission and fusion is critical in determining cell health (Wang and Youle, 2009). The involvement of ROS regulation, apoptosis, and AMPK-mediated mitochondrial dynamics in RA pathogenesis and treatment warrants further investigation.

Cornus officinalis Sieb. et Zucc, also known as Corni Fructus, is a plant traditionally used to treat tinnitus, impotence, and excessive urination. It has exhibited anti-oxidant (Lee et al., 2006), anti-diabetic (Yamahara et al., 1981), and anti-tumorigenic (Chang et al., 2004) effects. The active compounds of Cornus officinalis include malic acid, ursolic acid, and loganin (Endo and Taguchi, 1973; Czerwinska and Melzig, 2018; Dong et al., 2018). Among these, the relative content of loganin and ursolic acid are often evaluated in assessing the quality of Cornus officinalis. In particular, ursolic acid inhibited the release of inflammatory factors, repaired the balance between Th1/Th17, and suppressed matrix metalloproteinase production in an *in vivo* model of type II collagen-induced arthritis (Lee et al., 2017). Paeonia lactiflora Pall. has also demonstrated effectiveness in RA treatment. Paeoniflorin is one of the main active components of Paeonia lactiflora and has shown a variety of anti-inflammatory and immunomodulatory effects (Zhang and Wei, 2020). Taken together, both Cornus officinalis and Paeonia lactiflora have shown promising effects in RA treatment, but the specific molecular mechanism behind their therapeutic action have not been studied in-depth.

Herein, we explored the medicinal value of Cornus officinalis and Paeonia lactiflora in RA treatment using a rat model of collagen-induced arthritis (CIA). We hypothesized that the combination of Cornus officinalis and Paeonia lactiflora can effectively inhibit the release of oxidative stress-related and inflammatory factors during RA, therein ameliorating the symptoms and suppressing the progression of RA. We further propose that the underlying mechanisms may be related to the regulation of apoptosis in synovial tissues, and we investigated the potential involvement of AMPK-mediated mitochondrial dynamics in the therapeutic action of the two drugs.

## Materials and Methods

### CIA Induction and Drug Treatment

Commercially available Cornus officinalis (200309) and Paeonia lactiflora (200101) were purchased from Zhejiang Chinese Medical University Medical Pieces Ltd. (Hangzhou, China), ursolic acid (DX0019) and paeoniflorin (DS0070) were purchased from Desite (Chengdu, China), and dexamethasone (D1756) was purchased from Sigma-Aldrich (St. Louis, MO, United Ststes). To prepare the drugs for gavaging, each compound was dissolved in sterilized water at specified concentrations, given that the drugs were administered at 10ml per kg of animal body weight. All animal experiments were performed at Wuhan Myhalic Biotechnology Co., Ltd. (Wuhan, China). The experimental protocol was approved by the institutional review board of the Model Animal Research Institute at Wuhan Myhalic Biotechnology Co., Ltd. and adhered to the guidelines for animal care and use (approval number: HLK-20190418–01). Male specific-pathogen-free Sprague-Dawley rats weighing 200 ± 20 g were acquired from China Three Gorges University. The rats were housed in a facility with 50–60% relative humidity at 25°C. Before the experiment, the rats adaptively fed for seven days, where they were allowed free access to food and water. The CIA model was established following previously reported methods (Jia et al., 2014; Xu et al., 2018), with three rats in each group. Control rats were not treated in any way. To induce CIA, 10mg of type II collagen (PAB43878, Bioswamp) was mixed with 0.01M acetic acid and emulsified with an equal volume of Freund’s complete adjuvant to a final collagen concentration of 2mg/ml. On day 0, experimental rats were intradermally injected with 0.1ml of emulsified collagen II in the right hind toe. Boost immunization was induced after seven days with an additional intradermal injection of 0.1ml of emulsified collagen II in the right hind toe. Drug treatment began after another seven days (14 days of CIA induction) by gavaging the rats daily with the following drug doses: Cornus officinalis (COR, low/L dose: 1.68 g of drug per kg of rat body weight per day (g/kg/d); medium/M dose: 3.36 g/kg/d; high/H dose: 6.27 g/kg/d), Paeonia lactiflora (PAE, L: 3.36 g/kg/d; M: 6.27 g/kg/d; H: 13.44 g/kg/d), combination of COR and PAE, paeoniflorin (PF, 7.5 mg/kg/d) (Xu et al., 2018), ursolic acid (UA, 25 mg/kg/d) (Lee et al., 2017), or dexamethasone (DEX, 0.5 mg/kg/d) as a positive control. The doses of COR and PAE were determined based on the clinically administered doses of each drug, which range from 15 to 30 g/60 kg/d and 30–60 g/60 kg/d, respectively (60 kg being the normal adult human weight). The lower and higher ends of these ranges were taken to be the low (COR-L and PAE-L) and medium (COR-M and PAE-M) doses, respectively. The highest doses (COR-H and PAE-H) were determined by multiplying the medium dose by 2.5. These doses were then converted into corresponding drug doses for rats based on the literature (from human to rat: multiplied by 6.2) (Nair and Jacob, 2016).

### Evaluation of Toe Swelling and Arthritic Score

During the experimentation period, toe volume was measured by water displacement plethysmometry (Obiri et al., 2014; Zhang et al., 2016) on day 0, 4, 7, 12, 14, 24, 32, and 34. The degree of swelling was calculated using the following equation:

$$\%\;\mathrm{swelling=}\left(V_{x}-V_{0}\right)/V_{0}\times 100\%$$

Where V_x_ is the toe volume on day x and V_0_ is the initial toe volume measured on day 0 in the same drug treatment group. On the same days, the paw of the rats was observed macroscopically and the severity of arthritis was assessed. An arthritic score was given using the following scale (Wang et al., 2007): 0, no redness or swelling; 1, redness and swelling at the toe joints; 2, redness and swelling at the toes and toe joints; 3, redness and swelling in all areas below the ankle; 4, redness and swelling in all areas below the ankle, including the ankle joint.

### Sample Preparation

After 20 days of drug administration (34 days after initial CIA induction), the rats were sacrificed *via* an overdose of sodium pentobarbital. Whole blood was collected from the abdominal aorta of the rats and serum was separated by centrifugation. To isolate synovial tissues, the knee joint was cut open to expose the kneecap and separate the muscles. The synovial and fibrous layers of the joint capsule were separated using surgical scalpels, and synovial tissues were extracted. The right ankle joint was fixed in 4% paraformaldehyde, decalcified, and paraffin-embedded for histological examination.

### Hematoxylin and Eosin (HE) Staining

HE staining was performed to observe general joint tissue morphology. Decalcified and paraffin-embedded samples were frozen at −20°C and cut using a microtome at a thickness of 3 μm. The sections were fixed on microscope slides and deparaffinized, then washed with water for 2 min. The sections were stained with hematoxylin (G1140, Solarbio) for 5 min and residual dye solution was removed with running water. For differentiation, 1% hydrochloric alcohol was added to the sections for several seconds, and the sections were washed again with water. A bluing solution was added for 10 s and the sections were washed again with water. Next, the sections were stained with 0.5% eosin (E8090, Solarbio) for 3 min and washed briefly with distilled water. The sections were dehydrated in a graded concentration series of ethanol (80% for 30 s, 90% for 30 s, 100% for 2 s) and immersed twice in xylene for several seconds. The sections were then sealed and observed under an upright microscope (DM1000, Leica). Images of joint and bone tissue were acquired using Leica Application Suite. Each joint tissue and bone tissue section was given an inflammation score and bone destruction score, respectively, and the two scores were added to obtain the total pathology score. Inflammation was scored based on the following standards: 0, no inflammatory infiltration; 1, approximately 20 inflammatory infiltrates per field of view (400×); 2, 20 to 50 inflammatory infiltrates per field of view (400×); 3, more than 50 inflammatory infiltrates per field of view (400×). Bone destruction was scored based on the following standards: 0, normal; 1, small amount of cartilage loss and bone destruction is limited to isolated regions; 2, large areas of cartilage destruction and bone erosion caused by pannus; 3; complete destruction of joint structure.

### Terminal Deoxynucleotidyl Transferase dUTP Nick End Labeling (TUNEL) Staining

TUNEL staining was performed to evaluate tissue apoptosis in ankle joint samples using an *In Situ* Cell Death Detection Kit (11684817910, Roche). Decalcified and paraffin-embedded joint tissue samples were frozen at −20°C and cut using a microtome at a thickness of 3 μm. The sections were fixed on microscope slides and heated at 65°C for 1 h, then immersed twice in xylene for 15 min each. The deparaffinized sections were rehydrated in a graded concentration series of ethanol (100%, 95% s, 85%, and 75% for 5 min each) and washed with running tap water for 10 min. Then, the sections were incubated with proteinase K solution (P1120, Solarbio) at 37°C for 15 min and washed twice with PBS. TUNEL reaction solution was prepared by mixing 50 μl of enzyme solution with 450 μl of solvent (both included in the assay kit). After the PBS washes, the sections were incubated with 50 μl of TUNEL reaction solution in a humidified box in the dark for 1 h at 37°C. After three washes with PBS, 50 μl of POD solution (included in the assay kit) was added to each section, which were then incubated in a humidified box for 30 min at 37°C. The sections were washed three times with PBS and incubated with 50 μl of diaminobenzidine (DA1010–2, Solarbio) at room temperature for 10 min, after which they were washed again three times with PBS. Counterstaining was performed using hematoxylin, and the sections were dehydrated, transparentized, and sealed. Tissue apoptosis was observed under an upright microscope (DM1000, Leica) and analyzed using Leica Application Suite. Positive TUNEL staining representative of apoptotic cells is shown in dark brown.

### Biochemical Assays

The levels of cytokines in the serum of CIA-induced rats were evaluated using enzyme-linked immunosorbent assay. Commercial assay kits for TNF-α (RA30025), IL-1β (RA20020), IL-6 (RA20607), and IL-10 (RA20090) were obtained from Bioswamp and the assay was carried out by following the protocols specified by the manufacturer. Colorimetric assays were performed to detect the levels of oxidative stress-related factors in the serum and synovial tissues of CIA-induced rats. Commercial assay kits for malondialdehyde (MDA, A003–1), superoxide dismutase (SOD, A001–1), and nitric oxide (NO, A-013–2) were obtained from Nanjing Jiancheng Bioengineering Institute (Nanjing, China) and the assay was carried out by following the protocols specified by the manufacturers. All required reagents were included in the ELISA and colorimetry assay kits. For all biochemical assays, absorbance of the well plates was measured using a microplate reader (MK3, Finnpipette).

### Western Blot

Synovial tissues were homogenized and lysed using radioimmunoprecipitation assay buffer (JC9610, Gelatins) containing protease and phosphatase inhibitors. After complete lysis, the samples were centrifuged for 15 min at 12000 × g at 4°C, and the concentration of proteins in the supernatant was measured using a bicinchoninic acid assay kit (PN0120, G-CLONE). For western blot, sodium dodecyl sulfate-polyacrylamide gel electrophoresis was performed using 20 μg of denatured proteins from each sample. After electrophoresis, the proteins were transferred onto polyvinylidene fluoride membranes (16220405, DocLab), which were then blocked in 5% skim milk overnight at 4°C. Primary antibody incubation was performed for 1 h at room temperature with antibodies against the following proteins: Bax (1:1000, PAB32583, Bioswamp), Bcl-2 (1:1000, PAB33482, Bioswamp), cleaved caspase-3 (1:1000, 9661S, Cell Signaling Technology), cytochrome-c (1:1000, PAB30055, Bioswamp), dynamin-related protein 1 (Drp1, 1:1000, PAB33409, Bioswamp), phosphorylated Drp1 at serine 616 (pDrp1-Ser616, 1:1000, 4867S, Cell Signaling Technology), phosphorylated Drp1 at serine 637 (pDrp-Ser637, 1:1000, PA5-64821, eBioscience), mitochondrial fission process protein 1 (MTFP1, 1:1000, PAB42161, Bioswamp), mitofusin 2 (MFN2, 1:1000, PAB37988, Bioswamp), AMPK (1:1000, PAB30970, Bioswamp), and phosphorylated AMPK (pAMPK, 1:1000, ab23875, Abcam), with glyceraldehyde 3-phosphate dehydrogenase (GAPDH, 1:1000, PAB36296, Bioswamp) as a housekeeping control. The membranes were washed three times with PBS/Tween 20 for 5 min each and incubated with horseradish peroxidase-conjugated goat anti-rabbit IgG secondary antibodies (1:20000, SAB43714, Bioswamp) for 1 h at room temperature. After three washes with PBS/Tween 20 for 5 min each, the membranes were immersed in enhanced chemiluminescence solution and the protein bands were exposed using an automatic analyzer (JP-K900, Jiapeng, Shanghai, China). The gray values of the protein bands were measured using Tanon GIS software.

### Transmission Electron Microscopy

Synovial tissues were first fixed with 2.5% glutaraldehyde (30092436, Sinopharm) at 4°C for 30 min. After three washes with PBS for 10 min each, the tissues were fixed with 1% osmium acid (18,456, TedPella) for 1 h and washed again three times with PBS for 10 min each. To dehydrate the sample, the synovial tissues were sequentially immersed in ethanol at increasing concentrations (50, 70, 80, and 90%) for 5 min at each concentration, then in 90% acetone for 5 min and twice in 100% acetone for 4 min each. For soaking, the tissues were semi-immersed in a solution of acetone and epoxy resin 812 (1:1 ratio) at 40°C for 6 h and then fully immersed in pure epoxy resin 612 at 40°C for 4 h. After fixation, dehydration, and soaking, the tissues were embedded in a plastic plate and placed in an oven for polymerization for 4 h at 40°C, 2 h at 50°C, and 12 h at 90°C. The embedded samples were sectioned using an ultramicrotome at 60 nm. The sectioned samples were dyed in uranyl acetate (1005–25 g, Merck) in the dark for 20 min and washed three times with double-distilled water. After drying, the samples were stained with lead citrate (1101125, SPI) in the dark for 15 min. Excess lead residue was washed away with double-distilled water. The samples were blotted dry with filter paper and observed under a transmission electron microscope (HT7700, Hitachi).

### Statistical Analysis

Statistical analysis was performed using OriginPro 8.0. All data are presented as the mean ± standard deviation (*n* = 3). The significance of differences between means among more than two groups was evaluated by the one-way analysis of variance with Tukey’s post-hoc test. Statistical significance was defined as *p* < 0.05.

## Results

### Construction and Validation of CIA Model in Sprague-Dawley Rats

**FIGURE 1:**
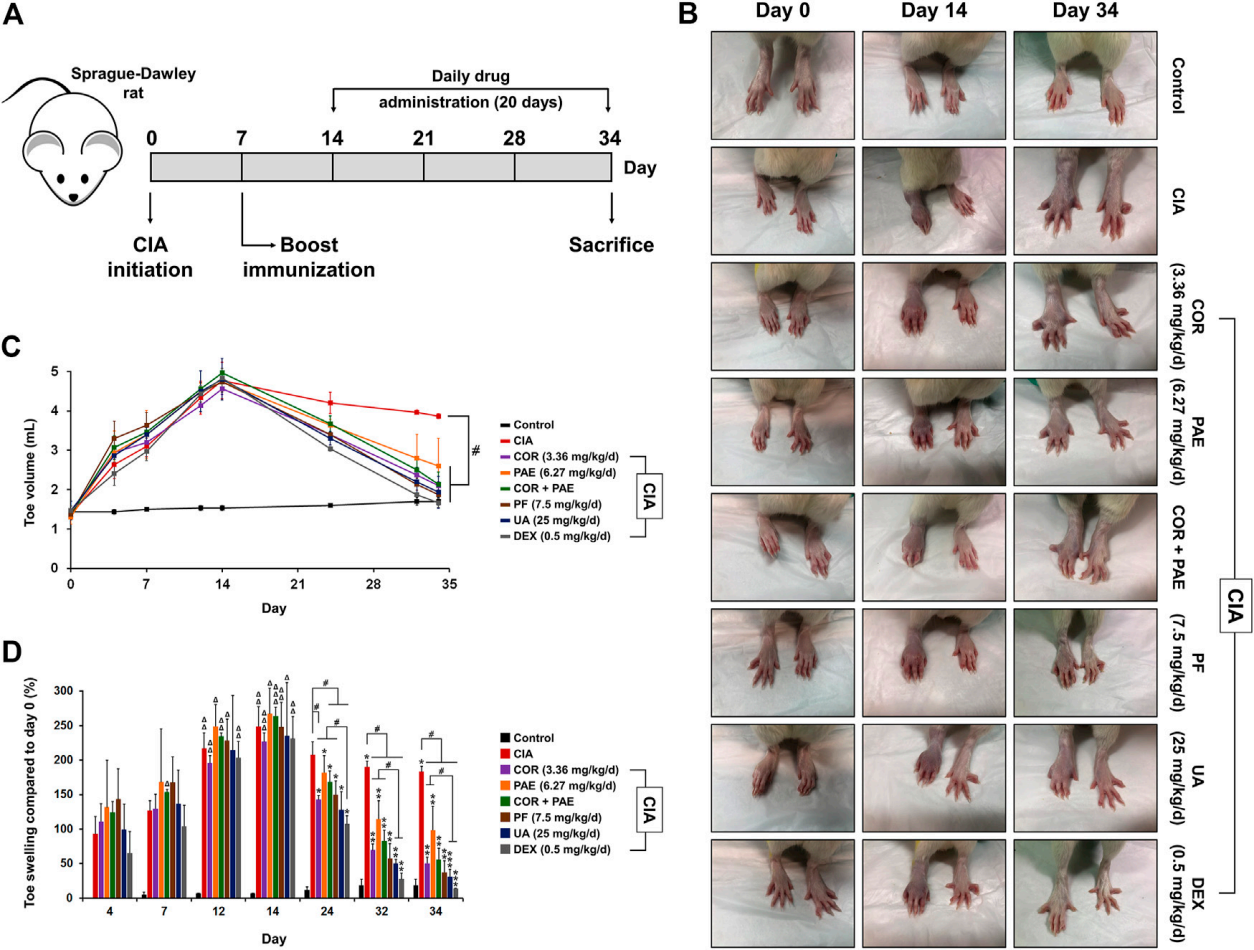
Validation of CIA model establishment. **(A)** To induce CIA, Sprague-Dawley rats were given an initial injection of CFA containing collagen II (day 0). After 7 days, boost immunization was induced via CFA injection. Drug administration began on day 14 and continued daily for 20 days. The rats were sacrificed on day 34. **(B)** Macroscopic observation of toe swelling on day 0 (CIA initiation), day 14 (beginning of drug treatment), and day 34 (end of drug treatment). **(C)** Toe volume was measured on day 0, 4, 7, 12, 14, 24, 32, and 34. **(D)** Relative toe swelling was calculated using the following equation (V_x_–V_0_)/V_0_ × 100%, where V_x_ is the toe volume measured on day x and V_0_ is the initial toe volume of the respective drug treatment group measured on day 0. All data are expressed as the mean ± standard deviation (*n* = 3 rats). #*p* < 0.05; Δ, ΔΔ, and ΔΔΔ denote *p* < 0.05 compared to day 4, 7, and 12, respectively, in the same drug treatment group; *, **, and *** denote *p* < 0.05 compared to day 14, 24, and 32, respectively, in the same drug treatment group. Low, medium, and high doses of COR and PAE were administered, but the medium doses (COR-M and PAE-M) were selected as representatives for analysis. CIA: collagen-induced arthritis; COR: Cornus officinalis; PAE: Paeonia lactiflora; PF: paeoniflorin; UA: ursolic acid; DEX: dexamethasone.

The experimental scheme in Figure 1A demonstrates the method of establishing the CIA model in Sprague-Dawley rats. Emulsified type II collagen in Freund’s complete adjuvant was injected on days 0 (initiation) and 7 (boost immunization), and drug administration began on day 14 and continued for 20 consecutive days. CIA-induced rats were treated with 3.36 mg/kg/d Cornus officinalis (COR) or/and 6.27 mg/kg/d Paeonia lactiflora (PAE), 7.5 mg/kg/d paeoniflorin (PF, active compound in PAE), 25 mg/kg/d ursolic acid (UA, active compound in COR), or 0.5 mg/kg/d dexamethasone (DEX, positive control drug). Macroscopic observation of the paws of the rats (Figure 1B) demonstrated intense redness and swelling of the toes in all CIA-induced rats on day 14. By day 34, the toes of untreated CIA-induced rats still showed redness and swelling, but those of drug-treated rats recovered to a large extent. In particular, the appearance of the toes of rats treated with paeoniflorin, ursolic acid, and DEX seemed to closely resemble that of Control rats, indicating the therapeutic efficacy of these treatments. An arthritic score was assigned to each rat on specified days during the experimental period as an indication of the severity of arthritis based on the pre-defined criteria (Table 1). Consistent with the macroscopic observations, CIA induced severe redness and swelling by day 14, but drug treatment alleviated these symptoms to varying degrees, with paeoniflorin, ursolic acid, and DEX showing the best effect among the tested drugs.

**TABLE 1:** Evaluation of arthritic score in CIA-induced rats (*n* = 3). Arthritic score was assessed using the following scale: 0, no redness or swelling; 1, redness and swelling at the toe joints; 2, redness and swelling at the toes and toe joints; 3, redness and swelling in all areas below the ankle; 4, redness and swelling at in all areas below the ankle, including the ankle joint. COR: Cornus officinalis; PAE: Paeonia lactiflora Pall.; PF: paeoniflorin; UA: ursolic acid; DEX: dexamethasone.

		Day 0	Day 4	Day 7	Day 12	Day 14	Day 24	Day 32	Day 34
	Control	0 ± 0	0 ± 0	0 ± 0	0 ± 0	0 ± 0	0 ± 0	0 ± 0	0 ± 0
	CIA + CIA	0 ± 0	3 ± 0	3 ± 0	4 ± 0	4 ± 0	4 ± 0	4 ± 0	3 ± 0
	COR (3.36 mg/kg/d)	0 ± 0	3 ± 0	3 ± 0	4 ± 0	4 ± 0	3.33 ± 0.58	2.33 ± 0.58	2 ± 0
	PAE (6.27 mg/kg/d)	0 ± 0	2.67 ± 0.58	3 ± 0	4 ± 0	4 ± 0	3 ± 0	3 ± 0	2 ± 1
	COR + PAE	0 ± 0	2.67 ± 1.15	3 ± 0	4 ± 0	4 ± 0	3.67 ± 0.58	2.67 ± 0.58	1.67 ± 0.58
	PF (7.5 mg/kg/d)	0 ± 0	3.33 ± 0.58	3.33 ± 0.58	4 ± 0	4 ± 0	3 ± 1	2 ± 1	1.33 ± 0.58
	UA (25 mg/kg/d)	0 ± 0	2 ± 0	3 ± 0	4 ± 0	4 ± 0	3.33 ± 0.58	2 ± 0	1.33 ± 1.15
	DEX (0.5 mg/kg/d)	0 ± 0	2.67 ± 0.58	2.67 ± 0.58	4 ± 0	4 ± 0	2.33 ± 0.58	2.67 ± 0.58	0.33 ± 0.58

During the experimental period, the rats were monitored and changes in toe volume, which were a sign of swelling, were measured on specified days (Figure 1C). Evidently, the peak of CIA induction, indicated by the greatest toe volume, appeared on day 14 (except for the Control group, which did not receive CIA induction), as drug treatment began thereafter. From day 14 to day 34 (end of experimental period), there was a gradual decrease in toe volume in all drug-treated rats. By day 34, the toe volume of all drug-treated rats was significantly lower than that of non-treated CIA-induced rats, almost reaching the level of Control rats. To further analyze the effect of CIA and drug treatment on toe healing in CIA-induced rats, the swelling rate was calculated (Figure 1D). All CIA-induced rats have achieved significant toe swelling compared to the beginning of the experiment by day 14. Upon drug treatment, toe swelling was gradually alleviated compared to that in non-treated rats (CIA), and swelling was significantly relieved by all drug treatments by day 34. Individual administration of COR seemed to be more effect than PAE in alleviating toe swelling, while combined administration of the two did not accentuate the effect of individual drug treatment. In addition, the effect of ursolic acid and DEX was time-dependent up to day 34, whereas that of COR, PAE, and paeoniflorin was time-dependent up to day 32.

### Cornus Officinalis and Paeonia Lactiflora Restored Normal Joint Morphology and Inhibited Joint Tissue Apoptosis in CIA-Induced Rats

**FIGURE 2:**
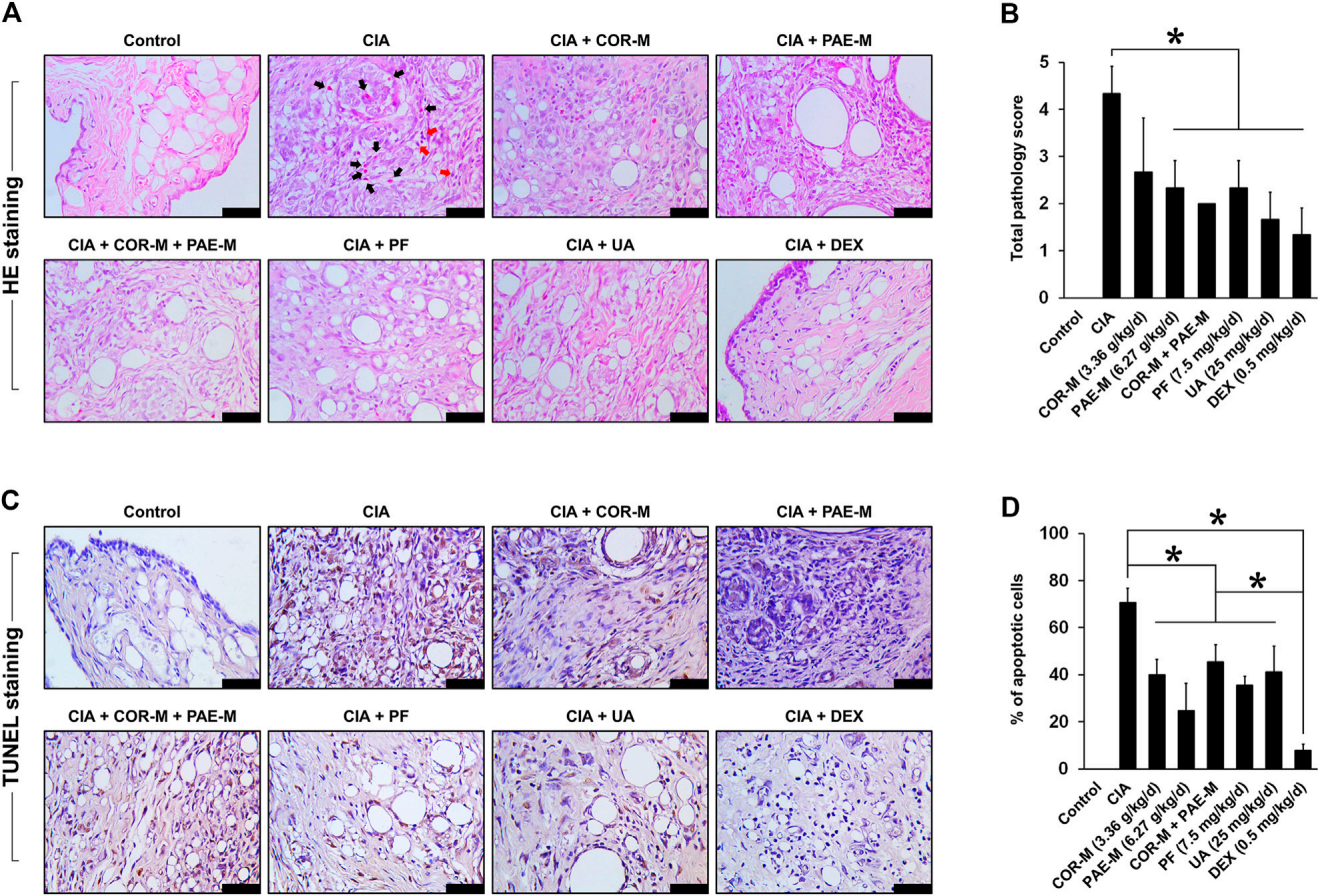
Effect of drug treatment on joint tissue morphology and apoptosis. Sprague-Dawley rats were induced by CIA for 14 days and subjected to daily drug administration at various doses for 20 consecutive days. **(A)** HE staining of the histopathology of joint tissues (scale bar, 50 μm). Eosinophils are indicated by black arrows and lymphocytes are indicated by red arrows. **(B)** Evaluation of inflammation score from the images of HE staining. **(C)** TUNEL staining of joint tissue apoptosis. Brown staining indicates apoptotic cells (scale bar, 50 μm). **(D)** Evaluation of the proportion of apoptotic cells from the images of TUNEL staining. All data are expressed as the mean ± standard deviation (*n* = 3 sections). *p < 0.05. Low, medium, and high doses of COR and PAE were administered, but the medium doses (COR-M and PAE-M) were selected as representatives for analysis. CIA: collagen-induced arthritis; COR: Cornus officinalis; PAE: Paeonia lactiflora; PF: paeoniflorin; UA: ursolic acid; DEX: dexamethasone.

The morphology of joint tissues was observed by HE staining after the animals were sacrificed on day 34 (Figure 2A). In the control rats, joint tissues exhibited normal morphology and did not show signs of inflammatory infiltration or fibrosis. In the untreated CIA-induced rats, however, hyperproliferation of synovial cells was observed. This was indicated by the filling of voids in the joint tissues, leading to synovial hyperplasia and thickening. In addition, we noticed areas of fibrous deposition and inflammatory infiltration, pointed out by black and red arrows (eosinophils and lymphocytes, respectively). Upon treatment using COR or/and PAE, these conditions seemed to be suppressed, as demonstrated by the reduction in synovial proliferation and hyperplasia. The effect of the individual active compounds paeoniflorin and ursolic acid appeared to be superior to that of COR and PAE, as joint tissue morphology most closely resembled those of control rats after paeoniflorin and ursolic acid treatment. In particular, inflammatory infiltration was cleared and synovial hyperplasia was remarkably alleviated. The inflammation scores (Figure 2B) are in agreement with the histological observations (results of bone destruction score are not shown), with CIA-induced rats showing the highest score. With each treatment, the inflammation score was significantly decreased (except for COR-M, where the difference was not significant).

Joint tissue apoptosis was examined by TUNEL staining after the animals were sacrificed on day 34 (Figures 2C,D). In the control rats, no apoptosis was detected in the joint tissues, but untreated CIA-induced rats showed a substantial amount of joint tissue apoptosis (approximately 70% of cells were apoptotic). Upon treatment using COR or/and PAE, tissue apoptosis was significantly suppressed compared to that in untreated CIA-induced rats, but the difference between treatment groups was not significantly. The individual active compounds paeoniflorin and ursolic acid exhibited similar effects in suppressing the apoptosis of joint tissues in CIA-induced rats, but the effect of the positive drug DEX was the most evident among all drugs tested.

### Cornus Officinalis and Paeonia Lactiflora Enhanced Anti-inflammatory Cytokine Secretion and Anti-oxidant Defense in CIA-Induced Rats

**FIGURE 3:**
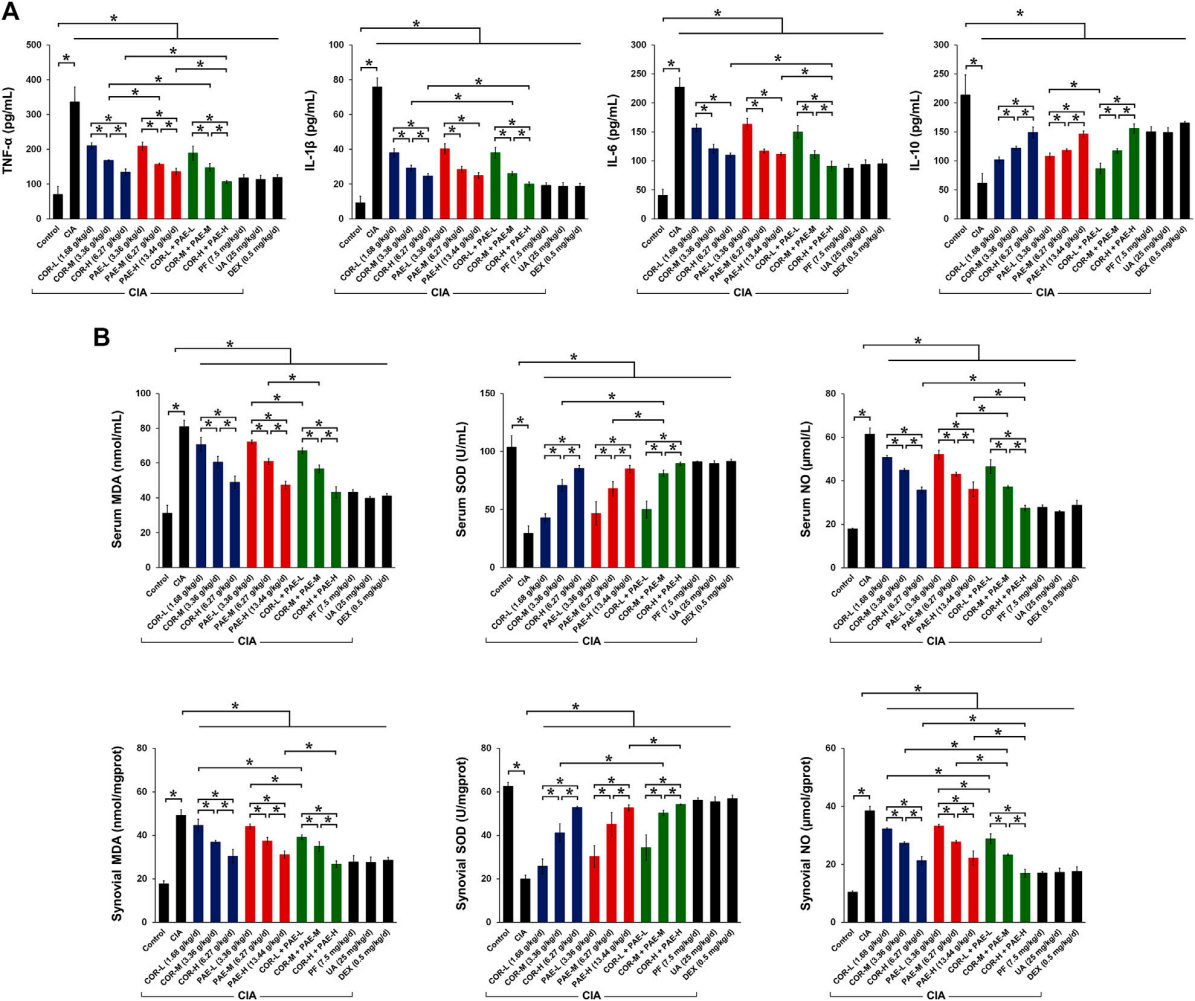
Effect of drug treatment on inflammatory cytokine secretion and oxidative defense in CIA-induced rats. Sprague-Dawley rats were induced by CIA for 14 days and subjected to daily drug administration at various doses for 20 consecutive days. **(A)** ELISA detection of the serum content of TNF-α, IL-1β, IL-6, and IL-10. **(B)** Biochemical detection of the content of MDA, SOD, and NO in the serum and synovial tissues. All data are expressed as the mean ± standard deviation (*n* = 3). *p < 0.05. Low, medium, and high doses of COR and PAE were administered, and dose response was analyzed. CIA: collagen-induced arthritis; COR: Cornus officinalis; PAE: Paeonia lactiflora; PF: paeoniflorin; UA: ursolic acid; DEX: dexamethasone; MDA: malondialdehyde; SOD: superoxide dismutase; NO: nitric oxide.

We then evaluated the effect of COR and PAE on the expression of inflammatory and oxidative stress-related indicators in CIA-induced rats. First, we examined the secretion of relevant cytokines in the serum of CIA-induced rats (Figure 3A). Compared to Control rats, CIA caused a significant increase in the secretion of pro-inflammatory factors (TNF-α, IL-1β, and IL-6) and a significant decrease in that of the anti-inflammatory factor IL-10. Both COR and PAE exerted a dose-dependent effect on cytokine secretion, suppressing inflammation by downregulating TNF-α, IL-1β, and IL-6 and upregulating IL-10. The combination of COR and PAE also showed a dose-dependent effect, but overall, the combined effect did not exceed that of individual COR or PAE administration. The active compounds paeoniflorin and ursolic acid seemed to exert similar effects as that of the positive control drug DEX in alleviating inflammatory response in CIA-induced rats. In terms of oxidative stress, the levels of factors associated with oxidative defense (MDA, SOD, and NO) were assessed in the serum and synovial tissues of CIA-induced rats (Figure 3B). The results showed that CIA induction led to a significant increase in oxidative stress, as shown by the upregulation of MDA and NO and the downregulation of the anti-oxidant SOD. Individual or combined administration of COR and PAE exerted an anti-oxidant effect, as demonstrated by the dose-dependent decrease in the levels of MDA and NO and increase in the level of SOD upon treatment. The active compounds paeoniflorin and ursolic acid seemed to exert similar effects as that of the positive control drug DEX in attenuating oxidative stress.

### Cornus Officinalis and Paeonia Lactiflora Regulated Synovial Apoptosis in CIA-Induced Rats

**FIGURE 4:**
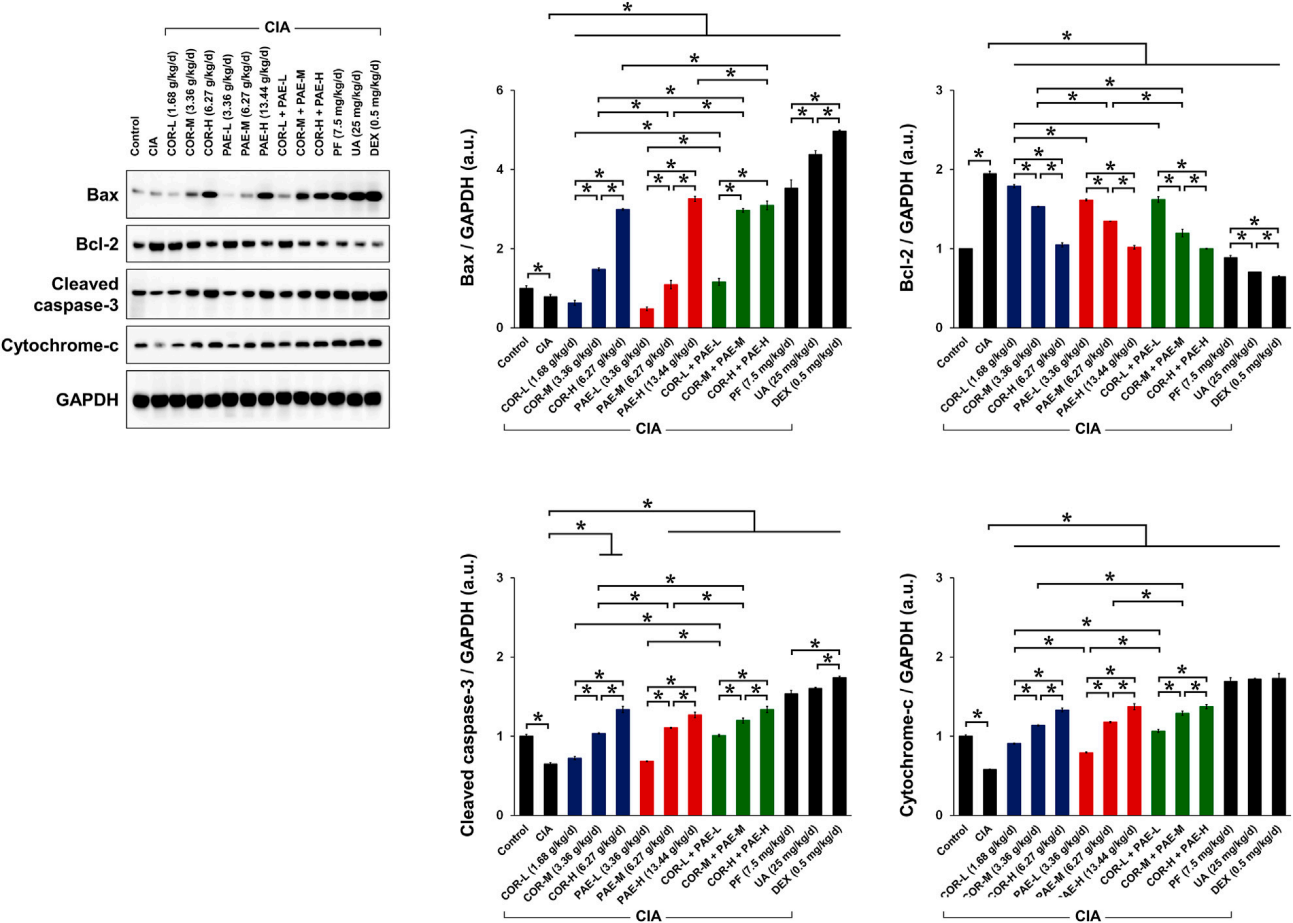
Effect of drug treatment on the expression of proteins associated with apoptosis in CIA-induced rats. Sprague-Dawley rats were induced by CIA for 14 days and subjected to daily drug administration at various doses for 20 consecutive days. Western blot detection and quantification of the expression of apoptosis-related proteins (Bax, Bcl-2, cleaved caspase-3, and cytochrome-c) in synovial tissues. All protein expression was normalized to that of GAPDH as a housekeeping control. All data are expressed as the mean ± standard deviation (*n* = 3). *p < 0.05. Low, medium, and high doses of COR and PAE were administered, and dose response was analyzed. CIA: collagen-induced arthritis; COR: Cornus officinalis; PAE: Paeonia lactiflora; PF: paeoniflorin; UA: ursolic acid; DEX: dexamethasone; GAPDH: glyceraldehyde 3-phosphate dehydrogenase.

To evaluate the effect of COR and PAE in synovial apoptosis, we examined the expression of relevant proteins in synovial tissues of control and CIA-induced rats. In terms of apoptosis (Figure 4), the expression of pro-apoptotic proteins Bax, cleaved caspase-3, and cytochrome-c was significantly downregulated in synovial tissues of CIA-induced rats compared to that in control rats. Conversely, the expression of the anti-apoptotic Bcl-2 was upregulated, collectively suggesting that synovial apoptosis was suppressed by CIA. Individual administration of COR or PAE in CIA-induced rats appeared to promote synovial apoptosis in a dose-dependent manner, and the combination of the two showed the same trend. Ursolic acid exerted a greater effect than paeoniflorin in affecting the expression of Bax and Bcl-2, but there was no difference between their effect on cleaved caspase-3 and cytochrome-c expression. Taken together, the results suggest that COR and PAE regulated apoptosis in synovial tissues of CIA-induced rats in a dose-dependent manner, and combination treatment exerted similar effects as those of individual treatment.

### Cornus Officinalis and Paeonia Lactiflora Promoted Mitochondrial Fission via AMPK Pathway in CIA-Induced Rats

**FIGURE 5:**
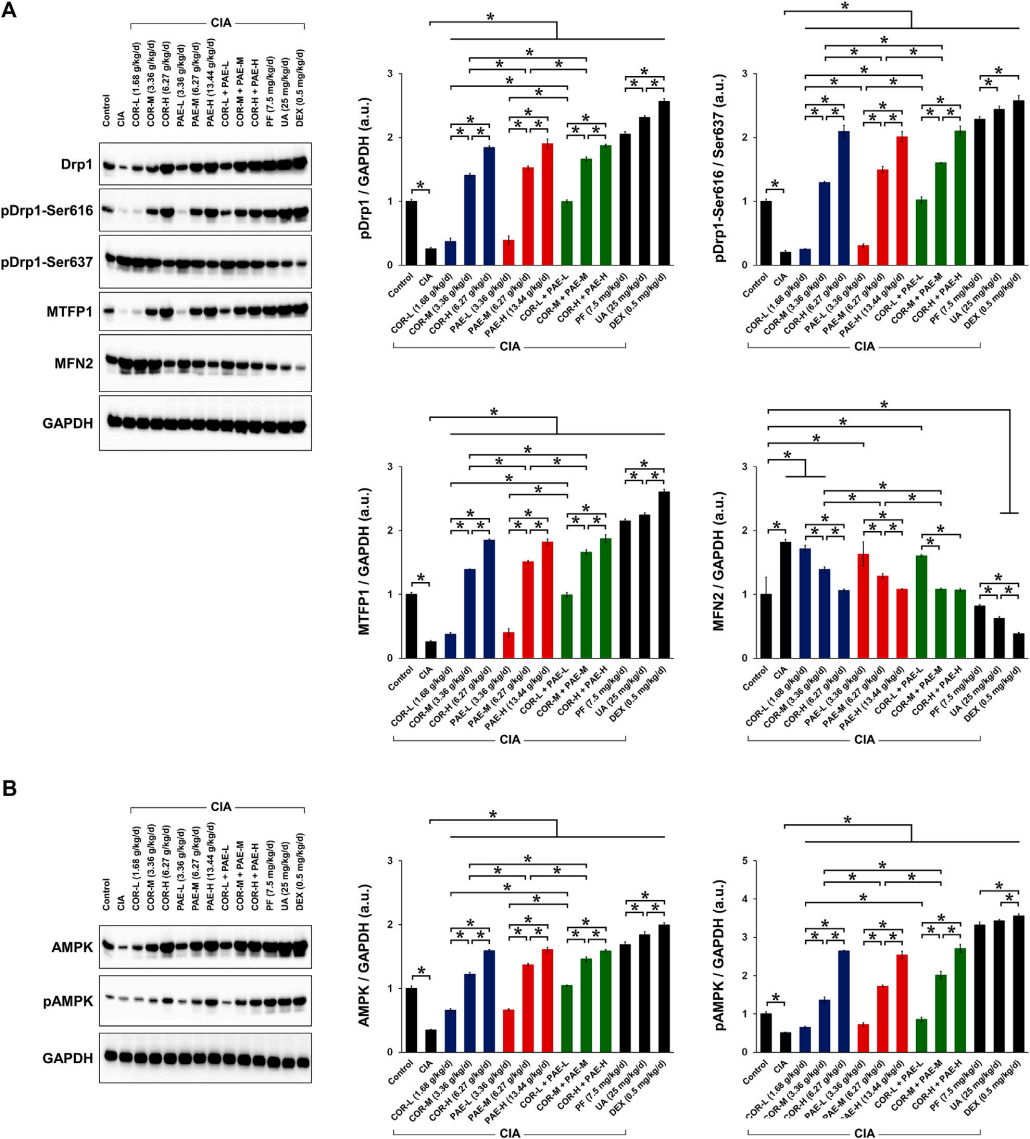
Effect of drug treatment on the expression of proteins associated with mitochondrial function and AMPK signaling in CIA-induced rats. Sprague-Dawley rats were induced by CIA for 14 days and subjected to daily drug administration at various doses for 20 consecutive days. **(A)** Western blot detection and quantification of the expression of mitochondrial function-related proteins (Drp1, Drp1 phosphorylation at Ser616 and Ser637, MTFP1, and MFN2) in synovial tissues. **(B)** Western blot detection and quantification of the expression of AMPK in its non-phosphorylated and phosphorylated form in synovial tissues. All protein expression was normalized to that of GAPDH as a housekeeping control. All data are expressed as the mean ± standard deviation (*n* = 3). *p < 0.05. Low, medium, and high doses of COR and PAE were administered, and dose response was analyzed. CIA: collagen-induced arthritis; COR: Cornus officinalis; PAE: Paeonia lactiflora; PF: paeoniflorin; UA: ursolic acid; DEX: dexamethasone; Drp1: dynamin-related protein 1; MTFN1: mitochondrial fission process protein 1; MFN2: mitofusin 2; AMPK: AMP-activated protein kinase; GAPDH: glyceraldehyde 3-phosphate dehydrogenase.

We next evaluated whether COR and PAE affected mitochondrial dynamics in synovial tissues after CIA induction by examining the expression of several mitochondria-related proteins (Figure 5A). These proteins include MTFP1, which is expressed as an indication of mitochondrial fission (Morita et al., 2017), and MFN2, which is expressed as an indication of mitochondrial fusion (Hu et al., 2020). We also looked at the phosphorylation of Drp1 at serine 616 and serine 637 as indications of mitochondrial fission activation and deactivation, respectively (Ko et al., 2016; Roe and Qi, 2018). Overall, the expression of MTFP1 and the ratio of pDrp1 (Ser616/Ser637) were significantly decreased in the synovial tissues of rats with CIA compared to those in control rats, whereas the expression of MFN2 was upregulated, signifying that mitochondrial fission was inhibited and fusion was promoted by CIA. This change was counteracted by drug treatment in CIA-induced rats. Specifically, individual administration of COR or PAE in CIA-induced rats promoted mitochondrial fission and suppressed fusion, as demonstrated by the dose-dependent increase in MTFP1 expression and pDrp1 (Ser616/Ser637) ratio and the decrease in MFN2 expression. Furthermore, the combination of the two showed the same trend, but the combined effect did not seem to be much stronger than the individual effects of the drugs. The active compounds ursolic acid and paeoniflorin exerted stronger effects in promoting mitochondrial fission than did individual or combined administration of COR and PAE, and the effect of ursolic acid was overall stronger than that of paeoniflorin at the administered doses.

The activation of AMPK is essential in facilitating mitochondrial fission and maintaining mitochondrial function. We examined the phosphorylation of AMPK in the synovial tissue of CIA-induced rats and investigated the effect of COR and PAE therein (Figure 5B). Both total and phosphorylated AMPK expression were downregulated in synovial tissues after the rats were induced by CIA. However, total AMPK expression and AMPK phosphorylation were restored by COR and PAE, whether administered individually or in combination, in a dose-dependent manner. The respective active compounds ursolic acid and paeoniflorin exerted even stronger effects in promoting AMPK phosphorylation. The results indicate that mitochondrial fission, which may contribute to mitochondrial dysfunction and lead to synovial tissue apoptosis, was promoted by drug administration. These findings are consistent with the previous results on apoptosis and mitochondrial function.

**FIGURE 6:**
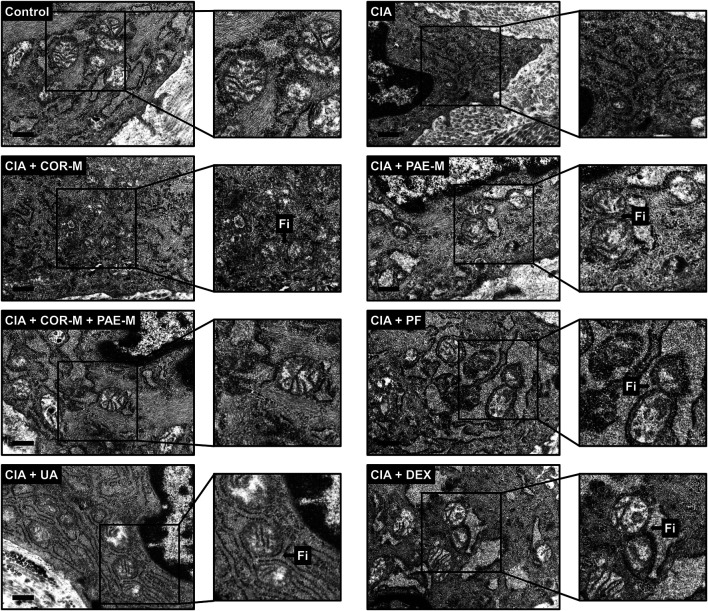
Transmission electron microscopy of mitochondrial morphology in synovial tissues. Sprague-Dawley rats were induced by CIA for 14 days and subjected to daily drug administration at various doses for 20 consecutive days. Selected areas in the microscopic images were magnified to show details of mitochondrial structure (scale bar, 500 nm). Arrows marked by “Fi” indicate areas where mitochondrial fission has occurred. Low, medium, and high doses of COR and PAE were administered, but the medium doses (COR-M and PAE-M) were selected as representatives for analysis. CIA: collagen-induced arthritis; COR: Cornus officinalis; PAE: Paeonia lactiflora; PF: paeoniflorin; UA: ursolic acid; DEX: dexamethasone.

To complement the results of western blot, we performed transmission electron microscopy to observe mitochondrial ultrastructure in synovial tissues (Figure 6). Mitochondria were oval and showed normal morphology in the synovial tissues of control animals, with clear and well-defined cristae. In rats subjected to CIA, the synovial mitochondria were smaller, their shape and structure were poorly defined, and cristae were barely recognizable. Mitochondrial structure remained poorly distinguishable upon COR-M treatment, but PAE-M seemed to promote mitochondrial fission (marked with "Fi" pointing to the junction where fission occurred) to a certain degree. When the CIA-induced rats were treated with a combination of COR-M and PAE-M, signs of mitochondrial fission became much clearer. Paeoniflorin and the positive control drug DEX seemed to have exerted the optimal effects in inducing mitochondrial fission, as the shape and ultrastructure of the mitochondria most clearly show a state of separation/division.

## Discussion

Traditional Chinese medicine has a history of treating rheumatoid conditions for thousands of years. The "multi-target" characteristics of traditional Chinese medicine have unique advantages in the prevention and control of complex diseases such as RA (Xia et al., 2020). The use of specific herbal or plant-based compounds in traditional Chinese medicine can alleviate symptoms and pathological damage and exert synergistic healing effects when combined with western medicine. Additionally, adverse reactions, drug resistance, and recurrence rate are lowered. Through modern medical theories and research methods (such as network pharmacology), Cornus officinalis and Paeonia lactiflora Pall have been identified as therapeutic candidates to treat RA. The findings of our study revealed that Cornus officinalis and Paeonia lactiflora, as well as their main active compounds ursolic acid and paeoniflorin, respectively, reduced synovial hyperplasia and inflammatory infiltration in joint tissues while promoting synovial apoptosis in a rat model of CIA. Each treatment also exerted anti-inflammatory and anti-oxidant defense by regulating the secretion of relevant cytokines and protein indicators. Furthermore, we demonstrated that the effects of the abovementioned compounds were related to synovial apoptosis, and that AMPK-mediated mitochondrial dynamics was involved in this process. In particular, Cornus officinalis, Paeonia lactiflora, ursolic acid, and paeoniflorin promoted synovial apoptosis. We propose that this occurs as a result of mitochondrial fission via enhanced activation of AMPK signaling in CIA-induced rats.

Overall, the effects of the individual compounds ursolic acid and paeoniflorin appeared to be stronger than those of Cornus officinalis and Paeonia lactiflora, respectively. As mentioned earlier, traditional Chinese medicine exerts multi-target properties, meaning that each type of herb or prescription is made up of a variety of biologically active compounds. Their role and potency may differ and each active compound may target different symptoms and biological anomalies. For example, some compounds may be more effective in regulating energy metabolism, while others may exert more potent effects against oxidative stress. In our case, we focused on studying ursolic acid and paeoniflorin, which are active compounds of Cornus officinalis and Paeonia lactiflora, respectively, that have previously shown strong anti-inflammatory and immunomodulatory activity (Lee et al., 2017; Zhang and Wei, 2020). When administered alone, the anti-inflammatory and immunomodulatory effects of ursolic acid and paeoniflorin are accentuated compared to when they are administered as part of an intricate complex of active compounds. In other words, their anti-inflammatory and immunomodulatory effects are isolated and concentrated, but as a component of Cornus officinalis and Paeonia lactiflora, respectively, their therapeutic role may be masked by other co-existing compounds. We suggest that this is the reason why individual administration of ursolic acid or paeoniflorin seemed to be more effective in alleviating RA than Cornus officinalis and Paeonia lactiflora themselves in this study. It is worth noting that we do not dismiss the utility and value of other active compounds but only focused on inflammation and immunomodulation here. The specific effects of other active compounds of Cornus officinalis (such as malic acid and loganin) and Paeonia lactiflora (such as albiflorin) will form the basis of future studies.

There is a delicate interplay between oxidative stress, inflammation, and mitochondrial function that governs the regulation of cellular apoptosis, which consequently affects a host of other downstream cellular processes. In the course of RA, fibroblast-like synoviocytes secrete a large number of pro-inflammatory factors such as TNF-α, IL-1β, IL-6, IL-8, IL-17A, and IL-23, which form a cytokine network to promote and maintain inflammation of the synovial membrane (McInnes and Liew, 2005). Neutrophils and activated macrophages secrete ROS when they are stimulated by excessive production of pro-inflammatory cytokines, making them a mediator of joint damage (Seven et al., 2008; Wendt et al., 2015). The accumulation of ROS may be one of the main causes of RA. Under oxidative stress conditions, overgeneration of ROS leads to lipid peroxidation and DNA fragmentation. This may cause structural and functional modifications of cellular proteins and extracellular components such as collagen and proteoglycans through processes including oxidation and nitration (Nash and Ahmed, 2015). In turn, the accumulation of oxidized cellular components and degradation products may increase synovial inflammation (Phull et al., 2018). In our study, Cornus officinalis and Paeonia lactiflora, as well as their main active compounds, inhibited inflammatory exacerbation and ROS generation in CIA-induced rats. This is evidenced by the decreased secretion of the pro-inflammatory factors TNF-α, IL-1β, and IL-6 and the increase in the anti-inflammatory factor IL-10, as well as the decreased secretion of pro-oxidative MDA and NO and the increase in the anti-oxidative SOD. These results exhibited the protective effects of the drugs against arthritic progression.

Mitochondria are the main source of cellular ROS and impaired balance in mitochondrial dynamics may result in cellular dysfunction, thereby leading to pathogenic conditions. It has been shown that hypoxia-induced mitochondrial dysfunction and oxidative damage cause disruptions to cellular metabolism, in turn promoting synovial invasiveness by exacerbating inflammatory response (Fearon et al., 2016). Mutations in mitochondrial DNA have also been identified as a pathological sign of RA (Da Sylva et al., 2005). In the maintenance of mitochondrial homeostasis, the AMPK signaling pathway plays an indispensable role by facilitating mitochondrial fission and recycling (Rabinovitch et al., 2017). Previously, ursolic acid has been shown to exhibit anti-tumor effects against human bladder cancer by activating AMPK signaling (Zheng et al., 2012), and paeoniflorin demonstrated protective effects against ischemia/reperfusion via AMPK activation (Wen et al., 2019). Herein, we showed that Cornus officinalis and Paeonia lactiflora, as well as their main active compounds, regulated apoptosis by upregulating Bax, cleaved caspase-3, and cytochrome-c and downregulating Bcl-2 via the activation of AMPK in CIA-induced rats. These findings have critical implications in elucidating the molecular basis of RA as they not only establish the importance of apoptosis control in RA development, but also point to AMPK as a potential therapeutic target that could be applied in treatment schemes.

Our study is subject to several limitations that need to be addressed in future research. Of primary interest are the specific gene and protein targets of Cornus officinalis, Paeonia lactiflora, ursolic acid, and paeoniflorin pertaining to RA, which were not explored in this study. The identification of such targets requires extensive investigation using tools such as bioinformatics and network pharmacology, which will be carried out as a follow-up. As the multi-target feature of traditional Chinese medicine is one of its unique characteristics, the effect of other active compounds of Cornus officinalis and Paeonia lactiflora on RA may also provide clues to their mechanisms of action. Bound by restrictions in material acquisition and laboratory conditions, we were unable to study compounds beyond ursolic acid and paeoniflorin here, but other active compounds will form the basis of future research. Additionally, the particular involvement of the AMPK signaling pathway in RA pathogenesis and the effect of ursolic acid and paeoniflorin needs to be confirmed using inhibitors of AMPK. Finally, this study lacks investigation on the effect of ROS and mitochondrial dysfunction on energy metabolism in RA. Future research will focus on this aspect and elucidate whether Cornus officinalis and Paeonia lactiflora (as well as their active compounds) also regulate energy metabolism to ameliorate RA.

## Conclusion

We showed here that Cornus officinalis, Paeonia lactiflora, ursolic acid, and paeoniflorin exhibited clear therapeutic effects against RA in an *in vivo* model of CIA. In particular, the abovementioned drugs promoted synovial apoptosis, favored mitochondrial fission, and enhanced the activation of AMPK signaling in CIA-induced rats. Overall, the effects of ursolic acid and paeoniflorin appeared to be stronger than those of Cornus officinalis and Paeonia lactiflora, respectively. This research highlights the potential of traditional Chinese medicine in the discovery of new targets and development of treatment strategies for RA, contributing to the advanced clinical management of RA.

## Data Availability

The raw data supporting the conclusions of this article will be made available by the authors, without undue reservation.
